# Numerical study of natural convection in a horizontal cylinder filled with water-based alumina nanofluid

**DOI:** 10.1186/s11671-015-0847-x

**Published:** 2015-03-19

**Authors:** Xiangyin Meng, Yan Li

**Affiliations:** Research Group of Offshore Engineering, School of Marine Science and Technology, Armstrong Building, Newcastle University, Newcastle upon Tyne, England NE1 7RU UK; Laboratory of Molecular Modeling and Design, State Key Laboratory of Molecular Reaction Dynamics, Dalian Institute of Chemical Physics, Chinese Academy of Sciences, 457 Zhongshan Road, Dalian, Liaoning 116023 China

**Keywords:** 44.05. + e, Nanofluid, Natural heat convection, OpenFOAM, Temperature-dependent solver

## Abstract

Natural heat convection of water-based alumina (Al_2_O_3_/water) nanofluids (with volume fraction 1% and 4%) in a horizontal cylinder is numerically investigated. The whole three-dimensional computational fluid dynamics (CFD) procedure is performed in a completely open-source way. Blender, enGrid, OpenFOAM and ParaView are employed for geometry creation, mesh generation, case simulation and post process, respectively. Original solver ‘buoyantBoussinesqSimpleFoam’ is selected for the present study, and a temperature-dependent solver ‘buoyantBoussinesqSimpleTDFoam’ is developed to ensure the simulation is more realistic. The two solvers are used for same cases and compared to corresponding experimental results. The flow regime in these cases is laminar (Reynolds number is 150) and the Rayleigh number range is 0.7 × 10^7^ ~ 5 × 10^7^. By comparison, the average natural Nusselt numbers of water and Al_2_O_3_/water nanofluids are found to increase with the Rayleigh number. At the same Rayleigh number, the Nusselt number is found to decrease with nanofluid volume fraction. The temperature-dependent solver is found better for water and 1% Al_2_O_3_/water nanofluid cases, while the original solver is better for 4% Al_2_O_3_/water nanofluid cases. Furthermore, due to strong three-dimensional flow features in the horizontal cylinder, three-dimensional CFD simulation is recommended instead of two-dimensional simplifications.

**Electronic supplementary material:**

The online version of this article (doi:10.1186/s11671-015-0847-x) contains supplementary material, which is available to authorized users.

## Background

Nanofluid is a suspension containing a certain quantity of nanoscaled solid particles in a conventional cooling liquid, such as water and ethylene glycol [1]. Nanofluid shows considerably better heat transfer performance than single-phase mediums due to particle’s Brownian motion and interaction [2,3]. Furthermore, because of the ultra-small particle size, nanofluid is also remarkably better than normal multi-phase fluid to eliminate erosion and clogging problems in micro channels [4,5]. Recently, nanofluid is increasingly used in natural convection applications for wide areas [6], such as electronic cooling, heat exchangers, boilers, nuclear reactor systems and energy storage devices [7].

To gain a better understanding of nanofluid natural heat convection, many studies have been carried out in both experimental and numerical ways during the past decade [8-10]. However, some apparently different conclusions can be found in those experimental and numerical investigations [11]. Briefly about nanofluid natural heat convection, deterioration was usually illustrated by experimental studies, while enhancement was always reported by numerical studies.

By experimental study, Putra et al. [12] found heat transfer deterioration in Al_2_O_3_/water and water-based copper oxide (CuO/water) nanofluids (with volume fraction 1% and 4%). They ascribed the possible reasons to particle-fluid slip and nanoparticle sedimentation. A similar observation was also reported by Wen and Ding [13]. In their experiments, 0.19% ~ 0.57% water-based titanium oxide (TiO_2_/water) nanofluids had a lower natural convective heat transfer coefficient than pure water, and the deterioration increased with volume fraction. They supposed the convection induced by particle concentration difference to be a possible reason. Li and Peterson [14] reported a natural heat convection deterioration in 0.5% ~ 6% Al_2_O_3_/water nanofluids. They inferred the reason could be the nanoparticle’s Brownian motion smoothing the temperature gradient leading to the delay of natural convection. Also, higher viscosity of nanofluids could also induce such an effect. Ni et al. [15] reported deteriorated natural convection after their experiments for 1.08% Al_2_O_3_/water nanofluid in a Rayleigh-Bénard configuration. They suggested that the significant decrease might be caused by the mass diffusion of nanoparticles. In Nnanna’s experiment [16], it was found that the presence of Al_2_O_3_ nanoparticles did not impede the water-free heat convection when the volume fraction was in the range of 0.2% ~ 2%. However, the heat convection declined due to increase of kinematic viscosity since the volume fraction was larger than 2%. Ho et al. [17] also reported up to 18% natural convective heat transfer enhancement in 0.1% Al_2_O_3_/water nanofluid, but degradation was found when the volume fraction was larger than 2%.

In numerical studies, some excellent molecular dynamic works have been carried out recently to analyse the possible heat transfer mechanisms between nanoparticle and fluid [18,19], such as Chiavazzo’s research on nanofins [20,21]. However, today, normal computational fluid dynamics (CFD) investigation is still playing a dominant role in this area. Khanafer et al. [22] found the water heat transfer rate in a two-dimensional enclosure could be substantially increased by adding more copper nanoparticles (*ϕ* was up to 20%). Oztop and Abu-Nada [23] found natural heat transfer enhancement by using Al_2_O_3_/water, TiO_2_/water and Cu/water nanofluids (*ϕ* was up to 20%) in two-dimensional rectangular enclosures with different aspect ratios. Furthermore, the enhancement was more pronounced at a low aspect ratio than at a high aspect ratio. Aminossadati and Ghasemi [24] reported that adding copper (Cu), silver (Ag), Al_2_O_3_ and TiO_2_ nanoparticles (*ϕ* was up to 20%) could improve cooling performance of pure water in a bottom-heated two-dimensional enclosure, especially when the Rayleigh number was low. Ghasemi and Aminossadati [25] reported a larger CuO/water nanofluid volume fraction (*ϕ* = 1% ~ 4%) led to Nusselt number enhancement in a two-dimensional triangular enclosure. Oueslati et al. [26] found nanofluid natural heat convection enhancement in a two-dimensional cavity when the volume fraction of Al_2_O_3_, TiO_2_ and Cu nanoparticles was lower than 5%. Ternik et al. [27] examined the heat transfer enhancement of water-based gold (Au), Al_2_O_3_, Cu and TiO_2_ nanofluids (*ϕ* was up to 10%) in a two-dimensional cavity. They indicated that the average Nusselt number was an increasing function of nanofluid volume fraction.

Actually, besides the above controversial conclusions from experimental and numerical investigations, dimension issue is another problem that should be noticed. In the past years, nearly all those experiments were performed in cylinders or tubes. They were of course three-dimensional investigations. However, previous numerical simulations were always performed for two-dimensional cases [22-27]. In fact, due to the gravity-induced force acting perpendicularly to the horizontal cylinder wall, the fluid movement in the cylinder could be apparently three-dimensional [12]. This could be an important factor influencing the internal flow and natural heat transfer behaviour in a horizontal cylinder. In previous publications, however, it is rare to see three-dimensional CFD investigations for correlative cases.

The aim of this study is to investigate Al_2_O_3_/water nanofluid natural heat convection in a horizontal cylinder by a three-dimensional CFD approach. For this, the numerical CFD package OpenFOAM [28] will be employed for case simulation in the present work. Furthermore, a new OpenFOAM solver will be developed to count in the possible impacts from temperature-dependent fluid property variation and make the numerical simulation more realistic.

## Methods

### Problem configuration

**Figure 1:**
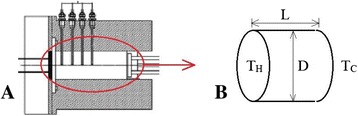
**Modelling prototype used in the present study.** Working session of Putra’s experimental device **(A)** [12]. Details: On 19th August 2014, Prof. Nandy Putra at the University of Indonesia authorized us to use his original figure which was published in [12]. Schematic experimental model, in which *D* is the cylinder diameter, *L* is the cylinder length, *T*_C_ is the temperature of the cooling wall and *T*_H_ is the temperature of the heating wall **(B)**.

In this investigation, a part of the experiment reported by Putra et al. [12] in 2003 is selected as the modelling prototype and will be repeated numerically. The working session of this experimental device (Figure 1A) is simplified to be an insulated cylinder with heating and cooling walls at the two ends (Figure 1B). The cylinder length and diameter are given as *L* = 0.04 m and *D* = 0.04 m, respectively. The ratio of cylinder length to diameter (*L*/*D*) is 1.0. A constant temperature *T*_C_ is given at the cooling end, and the input power is controlled at the heating end with temperature *T*_H_ to obtain the cases with different Rayleigh numbers. Non-permeable and non-slip boundaries are assumed for velocity. Blender (an open-source animation suite) [29] is used to create the three-dimensional geometry model. In this horizontal cylinder, natural heat convection of water and 1% and 4% Al_2_O_3_/water nanofluids will be investigated.

Recently, some researchers began to try different numerical methods and treated nanofluid as a multi-phase mixture, e.g. Eulerian [30,31] and Lagrangian approaches [32,33]. Even in some single-phase CFD simulations, the effect of nanoparticle-fluid slip began to be included, such as in Aminfar’s study [34]. However, there are still some arguments on the issue as to whether the multi-phase approach is better or not for nanofluids [35,36]. As the mechanisms of nanofluid thermal conductivity enhancement are still not very clear [37,38], the single-phase method is still used in the present work and Al_2_O_3_/water nanofluid is assumed to be a stable and homogeneous mixture as in traditional numerical investigations.

### Flow model

Since a stable purely laminar flow state could be eventually obtained in Putra’s experiment [12], original OpenFOAM solver ‘buoyantBoussinesqSimpleFoam’ is selected for this steady-state, buoyant, incompressible fluid case. In this solver, there are three coupled partial differential equations to describe flow and heat transfer problems; they are mass equation (1), momentum equation (2) and energy equation (3) [39]:

1 $$ \mathit{\nabla}\cdot u=0 $$

2 $$ \rho \mathit{\nabla}\cdot (uu)=-\mathit{\nabla}\rho +\mathit{\nabla}\cdot \left(\mu \mathit{\nabla}u\right)+{g}_k $$

3 $$ \rho \mathit{\nabla}\cdot \left({c}_pTu\right)=\mathit{\nabla}\cdot \left(k\mathit{\nabla}T\right) $$

where *u*, *p*, *T*, *ρ*, *c*_*p*_, *k*, *μ* and *g*_*k*_ indicate the velocity, pressure, temperature, density, specific heat capacity, thermal conductivity, dynamic viscosity and body force, respectively. ‘∇’ and ‘∇’ indicate divergence and gradient operations, respectively. Body force *g*_*k*_ is evaluated by Boussinesq approximation (4) [40]:

4 $$ {g}_k=\rho \left[1.0-\beta \left(T-{T}_{\mathrm{Ref}}\right)\right]g $$

where *β*, *T*_Ref_ and *g* are the thermal expansion coefficient, reference temperature and gravity, respectively.

Al_2_O_3_/water nanofluid density *ρ*_nf_ is evaluated by (5) [22,23]:

5 $$ {\rho}_{\mathrm{nf}}=\left(1-\varnothing \right){\rho}_{\mathrm{f}}+\varnothing {\rho}_{\mathrm{s}} $$

Al_2_O_3_/water nanofluid heat capacity *c*_*p*nf_ is evaluated by (6) [22,23]:

6 $$ {\rho}_{\mathrm{nf}}{c}_{p\mathrm{n}\mathrm{f}}=\left(1-\varnothing \right){\left(\rho {c}_p\right)}_{\mathrm{f}}+\varnothing {\left(\rho {c}_p\right)}_{\mathrm{s}} $$

where subscripts f, s and nf indicate fluid, solid and nanofluid, respectively.

Water properties are collected from a heat transfer textbook [41]. Density and specific heat capacity of Al_2_O_3_ nanoparticles are used as 3,970 kg m^−3^ and 765 J kg^−1^ K, respectively [23]. To ensure the simulation is more reliable, thermal conductivity *k* and kinetic viscosity *v* of 1% and 4% Al_2_O_3_/water nanofluids are collected from Das’s study [12,42] instead of prediction models. Based on regression analysis, thermal conductivity enhancement variation will be given by (7) ~ (9), while kinetic viscosity variation will be given by (10) ~ (12). For original solver ‘buoyantBoussinesqSimpleFoam’ , fluid properties are considered as constant for each case and evaluated at the mean of the heating and cooling wall temperatures (in Celsius degree).

7 $$ {k}_{\mathrm{water}}=0.0027\left(T-273\right)+0.9716 $$

8 $$ {k}_{1\%}=0.0028\left(T-273\right)+0.9672 $$

9 $$ {k}_{4\%}=0.0053\left(T-273\right)+0.977 $$

10 $$ {v}_{\mathrm{water}}=0.0002{\left(T-273\right)}^2-0.0305\left(T-273\right)+1.52 $$

11 $$ {v}_{1\%}=0.0003{\left(T-273\right)}^2-0.045\left(T-273\right)+1.96 $$

12 $$ {v}_{4\%}=0.0003{\left(T-273\right)}^2-0.0448\left(T-273\right)+2.16 $$

Based on ‘buoyantBoussinesqSimpleFoam’ , a new solver, ‘buoyantBoussinesqSimpleTDFoam’ , is developed to include possible impacts from fluid property variation due to temperature change. This temperature-dependent solver assumes that the fluid properties in a computational region are not uniform but decided by each volume cell’s temperature. During numerical simulation, strongly temperature-dependent fluid properties (e.g. thermal conductivity and viscosity) will be updated for each volume cell at each iteration after the energy equation has been solved.

Dimensionless parameter Nusselt number *Nu* is used to describe the fluid natural heat convection performance in those cases with different Rayleigh numbers *Ra*. They are defined in (13) and (14), respectively [22,23]:

13 $$ Nu=\frac{h{L}_{\mathrm{c}}}{k} $$

14 $$ Ra=\frac{\beta g\left({T}_{\mathrm{H}}-{T}_{\mathrm{C}}\right){L_{\mathrm{c}}}^3}{v\alpha } $$

where *h*, *L*_c_, *v* and *α* indicate the heat transfer coefficient, characteristic length, kinetic viscosity and thermal diffusivity, respectively. Convection heat transfer coefficient *h* is defined in (15) [22,23]:

15 $$ h=\frac{4Q}{\pi {D}^2\left({T}_{\mathrm{H}}-{T}_{\mathrm{C}}\right)} $$

where *Q* is the changeable input power, by which different Rayleigh numbers can be obtained for different cases.

### Numerical implementation and grid independence check

The set of three-dimensional coupled non-linear differential equations are discretized by control volume technique [43]. The semi-implicit method for pressure-linked equations (SIMPLE) algorithm [44] is employed in both ‘buoyantBoussinesqSimpleFoam’ and ‘buoyantBoussinesqSimpleTDFoam’ to solve Navier-Stokes equations. The spatial schemes for gradient, Laplacian and divergence are Gauss linear, Gauss linear corrected and Gauss linear schemes, respectively. During the iterative process, the absolute residuals of *u*, *p* and *T* are carefully monitored and convergence criteria for every parameter is restricted below 10^−6^.

**Figure 2:**
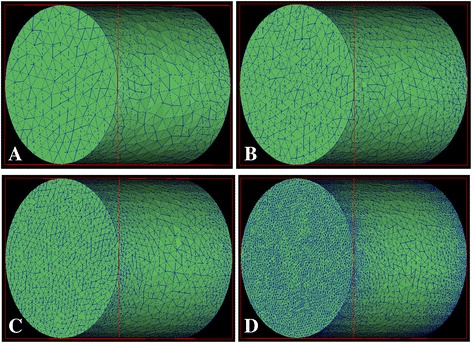
**Four mesh strategies for grid independence check:** Δ***d*** **= 4 mm (A), 3 mm (B), 2 mm (C) and 1 mm (D).**

An extensive testing procedure is carried out to guarantee a grid-independent solution. By enGrid (an open-source mesh generation package) [45], unstructured tetras are used to fill the three-dimensional cylinder. Four mesh strategies are generated for the grid independence check; their maximal cell edge lengths Δ*d* on heating and cooling walls are 4 mm (Figure 2A), 3 mm (Figure 2B), 2 mm (Figure 2C) and 1 mm (Figure 2D). To ensure simulation accuracy for near-wall regions, a non-uniform strategy is used to refine the mesh near heating and cooling walls. Original OpenFOAM solver ‘buoyantBoussinesqSimpleFoam’ is employed to test the four mesh strategies for a water case *Ra* = 10^8^. Since this solver has been validated in Corzo et al.’s work [46], a similar validation will not be repeated and the solver will be used directly.

**Figure 3:**
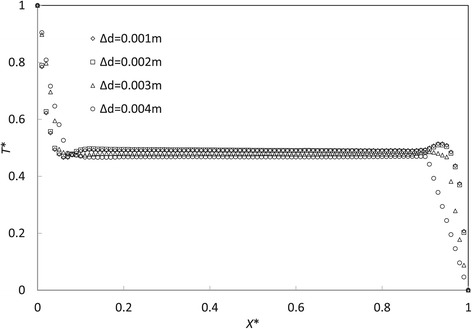
**Grid independence check.**

Dimensionless temperature T* and location X* are defined in (16) and (17) for the grid independence check, respectively. Results of T* and X* on the cylinder longitudinal central line are compared to find the most appropriate mesh strategy. By the comparison in Figure 3, mesh strategies Δ*d* = 2 mm and Δ*d* = 1 mm are found to predict nearly exactly the same results. This indicates the mesh strategy Δ*d* = 2 mm is good enough for the present study. However, the strategy Δ*d* = 1 mm is eventually selected to capture even more detailed velocity and temperature features in near-wall regions. Compared to normal two-dimensional simulations, the cell amount is considerably increased in this work. Although non-uniform grid strategies are employed to reduce the total cell number, there are still about 0.4 million cells used in the present work (Figure 2D).

16 $$ {T}^{*}=\frac{T-{T}_{\mathrm{C}}}{T_{\mathrm{H}}-{T}_{\mathrm{C}}} $$

17 $$ {X}^{*}=\frac{X}{L_{\mathrm{c}}} $$

## Results and discussion

**Figure 4:**
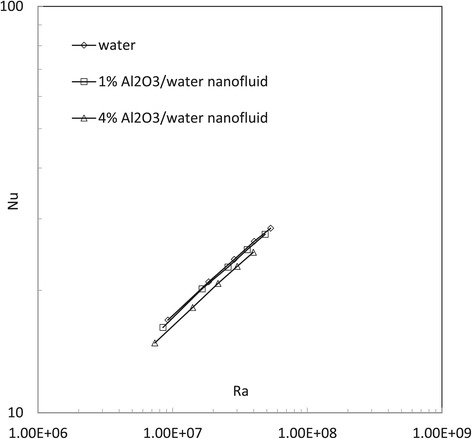
**Results of Nusselt number predicted by the solver ‘buoyantBoussinesqSimpleFoam’.**

**Figure 5:**
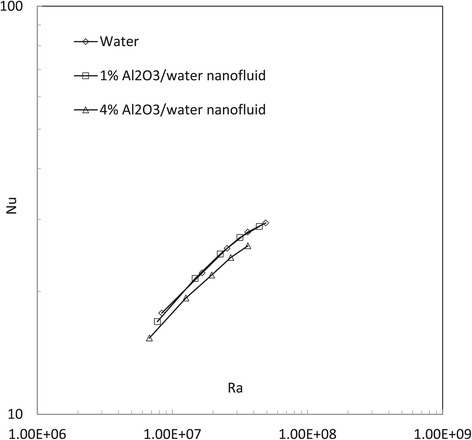
**Results of Nusselt number predicted by the solver ‘buoyantBoussinesqSimpleTDFoam’.**

**Figure 6:**
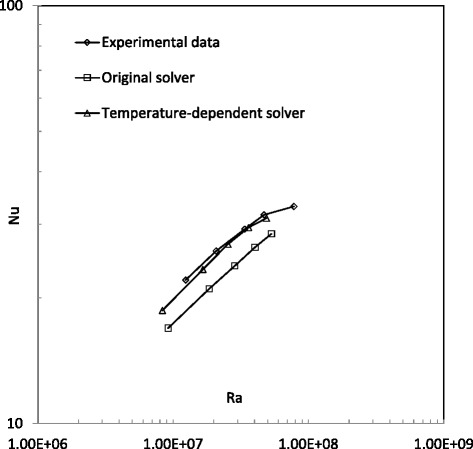
**Comparison of Nusselt number in water cases.**

**Figure 7:**
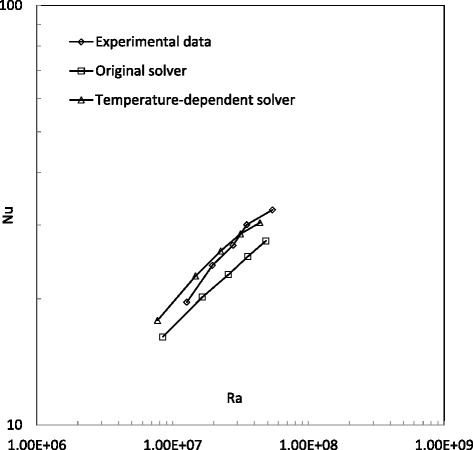
**Comparison of Nusselt number in 1% Al**_**2**_ **O**_**3**_ **/water nanofluid cases.**

**Figure 8:**
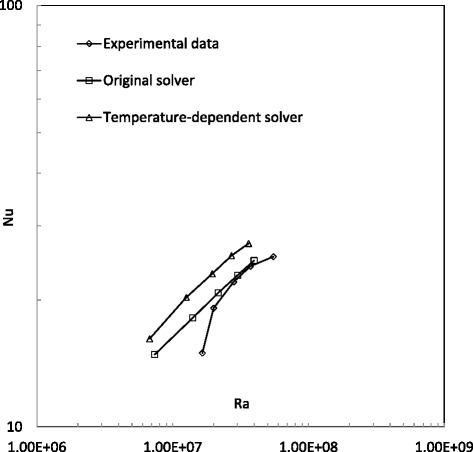
**Comparison of Nusselt number in 4% Al**_**2**_ **O**_**3**_ **/water nanofluid cases.**

In this study, natural heat convection of water and 1% and 4% Al_2_O_3_/water nanofluids is investigated numerically by both ‘buoyantBoussinesqSimpleFoam’ and ‘buoyantBoussinesqSimpleTDFoam’. For each fluid, five simulations are performed in the Rayleigh number range *Ra* = 10^7^ ~ 0.8 × 10^8^. Figures 4, 5, 6, 7 and 8 show the results of the average Nusselt number against the Rayleigh number in different cases. Basically, by both the present numerical study and previous experimental study, the natural Nusselt number of water and Al_2_O_3_/water nanofluids is found to increase with the Rayleigh number. However, some more information also can be found by further comprehensive comparisons in the present work.

Figure 4 shows the Nusselt number results predicted by the original solver ‘buoyantBoussinesqSimpleFoam’ for the cases of water and 1% and 4% Al_2_O_3_/water nanofluids. It can be found that 1% Al_2_O_3_/water nanofluid has no apparently different natural Nusselt numbers with water, but 4% Al_2_O_3_/water nanofluid has slightly lower Nusselt numbers. The possible reason is that although a nanofluid with a larger volume fraction has larger thermal conductivity, a larger volume fraction also induces larger fluid viscosity. The increased viscosity probably plays a predominant role to impede nanofluid natural heat convection.

Figure 5 shows the Nusselt number results predicted by the temperature-dependent solver ‘buoyantBoussinesqSimpleTDFoam’ for the cases of water and 1% and 4% Al_2_O_3_/water nanofluids. It can be found that 1% Al_2_O_3_/water nanofluid has no apparently different natural Nusselt numbers with water. But 4% Al_2_O_3_/water nanofluid has lower natural Nusselt numbers. Compared to Figure 4, the two solvers give a very similar conclusion: the nanofluid does not have a better natural convective heat transfer performance than the basefluid. This actually conforms to those experimental results [12,13], but in contradiction to some previous numerical conclusions [24,25]. For the difference between the present simulations and other numerical studies, the reason inferred is the possible impacts from the cylinder wall (in previous two-dimensional investigations, wall effect from the third direction could not be included to influence fluid flow and heat transfer).

Figure 6 shows the Nusselt number predictions from the original solver, temperature-dependent solver and experimental study for water cases. It can be found that the original solver ‘buoyantBoussinesqSimpleFoam’ predicts an apparently lower Nusselt number than the experimental study, but the results from the temperature-dependent solver ‘buoyantBoussinesqSimpleTDFoam’ have good agreement to experimental data, particularly when the Rayleigh number is larger than 2.0 × 10^7^. Figure 7 shows a similar comparison for 1% Al_2_O_3_/water nanofluid cases. It can be found that the original solver ‘buoyantBoussinesqSimpleFoam’ does not predict good values in the whole Rayleigh number range, but the results from the new solver ‘buoyantBoussinesqSimpleTDFoam’ begin to have good agreement with experimental data since the Rayleigh number is larger than 2.0 × 10^7^. By the comparisons in Figures 6 and 7, it can be concluded that the temperature-dependent solver ‘buoyantBoussinesqSimpleTDFoam’ is better for water and Al_2_O_3_/water nanofluids with a low volume fraction.

Figure 8 shows the Nusselt number predictions from the original solver, new solver and experimental study for 4% Al_2_O_3_/water nanofluid cases. It can be found that the predictions from the original solver ‘buoyantBoussinesqSimpleFoam’ begin to have good agreement with experimental data since the Rayleigh number is larger than 2.0 × 10^7^. However, the newly developed solver ‘buoyantBoussinesqSimpleTDFoam’ predicts a larger Nusselt number than the experimental study. For this phenomenon, nanoparticle sedimentation is thought to be a possible reason. This is also reported by some recent nanoparticle sedimentation observations [37,47]. Due to an improper nanoparticle dispersion method after fabrication, the nanofluid with a higher volume fraction will show a settlement layer quickly at the vessel bottom, leading to considerable deterioration of heat transfer performance. However, this mechanism is not included in the present single-phase CFD approach.

**Figure 9:**
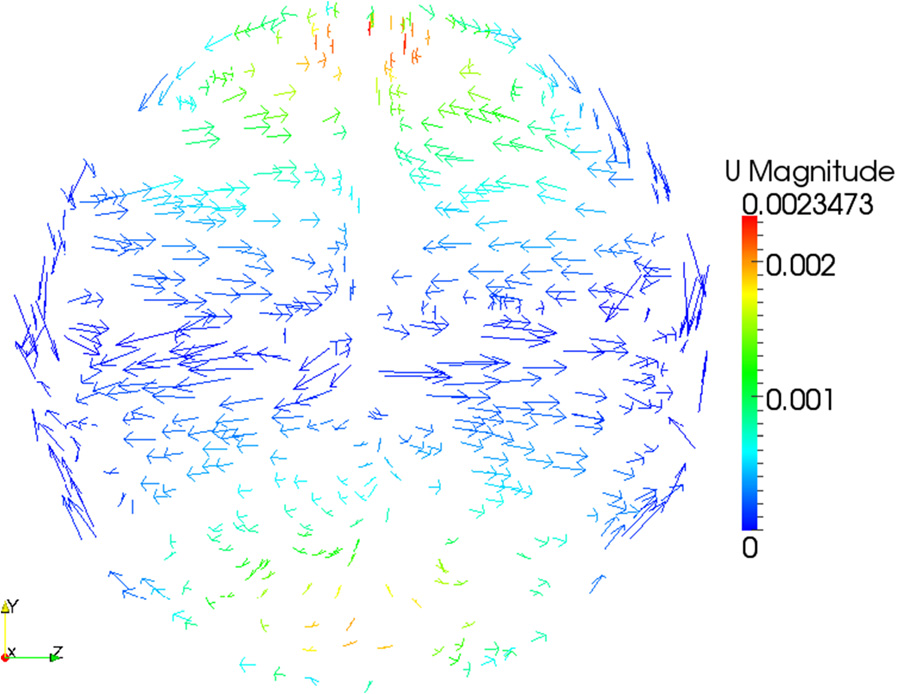
**Velocity features on cylinder cross section** ***X*** **= 0.01 m.**

**Figure 10:**
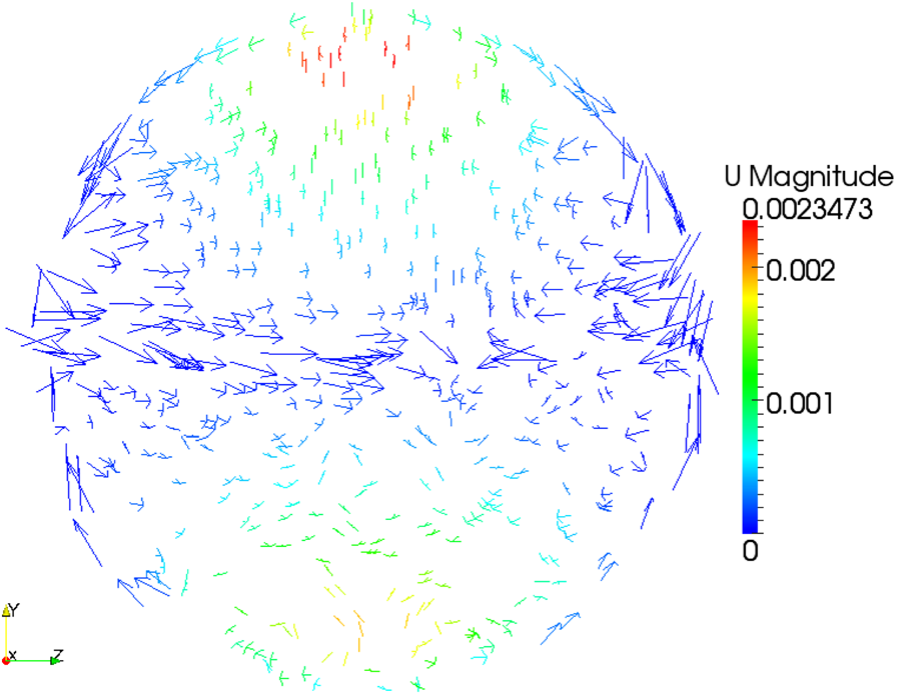
**Velocity features on cylinder cross section** ***X*** **= 0.02 m.**

**Figure 11:**
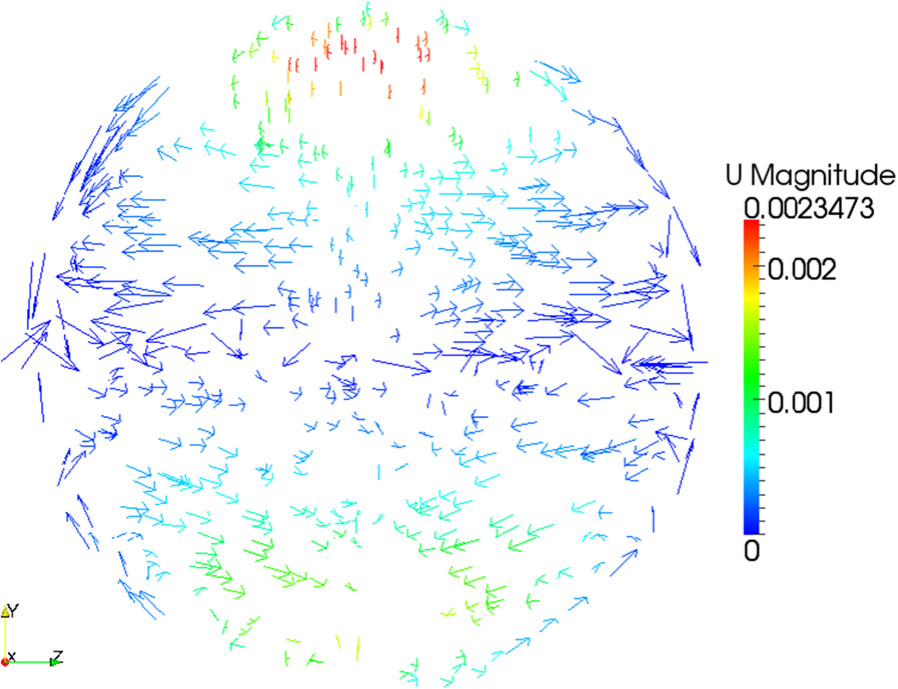
**Velocity features on cylinder cross section** ***X*** **= 0.03 m.**

More details of cylinder internal flow features are disclosed in this work. With the help of ParaView (an open-source visualization application) [48], Figures 9, 10 and 11 show the velocity vector features on three typical cylinder cross section positions - *X* = 0.01 m, *X* = 0.02 m and *X* = 0.03 m - for a 4% Al_2_O_3_/water nanofluid case with *Ra* = 4 × 10^7^. In the three figures, considerably horizontal and asymmetric velocity components can be observed, which indicate that the temperature-driven flow in the cylinder is actually different than that in broad rectangular ducts [49,50]. Therefore, due to the strong three-dimensional flow features, applying two-dimensional simplification and neglecting the possible impacts from the cylinder wall may be not so realistic. Briefly, for natural heat convection problems in a horizontal cylinder, a three-dimensional CFD simulation is assumed to be more reliable than two-dimensional simplifications [51].

## Conclusions

In this study, natural heat convection of Al_2_O_3_/water nanofluids in a horizontal cylinder (*L*/*D* = 1) is numerically investigated. The whole three-dimensional CFD process is performed in a completely open-source way. Blender, enGrid, OpenFOAM and ParaView are employed for geometry model creation, mesh generation, case simulation and post process, respectively. Natural heat convection of water and 1% and 4% Al_2_O_3_/water nanofluids is investigated by both the original OpenFOAM solver ‘buoyantBoussinesqSimpleFoam’ and the newly developed temperature-dependent solver ‘buoyantBoussinesqSimpleTDFoam’. Based on the obtained results in this investigation, several conclusions can be drawn as follows:

1. The natural convective Nusselt number of both water and Al_2_O_3_/water nanofluids in the horizontal cylinder (*L*/*D* = 1) increases with the Rayleigh number in the range of *Ra* = 0.7 × 10^7^ ~ 5 × 10^7^. With a given Rayleigh number, a larger nanofluid volume fraction induces a lower Nusselt number.
2. Numerical solvers in the OpenFOAM frame begin to give better agreements to experimental investigations since the Rayleigh number is larger than 2 × 10^7^. The temperature-dependent solver is better for water and 1% Al_2_O_3_/water nanofluid cases, but the original solver is better for 4% Al_2_O_3_/water nanofluid cases.
3. Due to strong three-dimensional flow features being observed, the three-dimensional CFD simulation is recommended for natural heat convection problems in a horizontal cylinder. This would be the best way to account for the possible impacts from the cylinder wall.

In a future study, a new OpenFOAM solver will be designed based on the present temperature-dependent one and treat nanofluid as a multi-phase mixture. Furthermore, it will be a transient solver instead of the currently steady-state one in this work. By applying proper mixture models, the interaction between nanofluid natural heat transfer and nanoparticle sedimentation will be observed (Additional file 1). This is necessary to figure out whether the nanoparticle sedimentation and nanoparticle-fluid slip have a remarkable impact on nanofluid natural heat convection in a horizontal cylinder.

### Nomenclature

- *c*_*p*_, Specific heat capacity (J kg^−1^ K^−1^)
- Δ*d*, Maximum cell edge length (m)
- *D*, Cylinder diameter (m)
- *g*, Gravity (m s^−2^)
- *g*_*k*_, Boussinesq body force (kg m^−2^ s^−2^)
- *h*, Heat transfer coefficient (W m^−2^ K^−1^)
- *k*, Thermal conductivity (W m^−1^ K^−1^)
- *L*, Cylinder length (m)
- *L*_c_, Characteristic length (m)
- $$ Nu=\frac{h{L}_{\mathrm{c}}}{k} $$, Nusselt number
- *p*, Pressure (kg m^−1^ s^−2^)
- *Q*, Input power (W)
- *u*, Velocity (m s^−1^)
- *T*, Temperature (K)
- *T*_C_, Temperature of cooling wall (K)
- *T*_H_, Temperature of heating wall (K)
- *T*_Ref_, Reference temperature (K)
- T*, Dimensionless temperature
- X*, Dimensionless position
- $$ Ra=\frac{\beta g\varDelta T{L_{\mathrm{c}}}^3}{v\alpha } $$, Rayleigh number

Greek symbols

- *α*, Thermal diffusivity (m^2^ s^−1^)
- *β*, Thermal expansion coefficient (K^−1^)
- *μ*, Dynamic viscosity (NS m^−2^)
- *v*, Kinetic viscosity (m^2^ s^−1^)
- *ρ*, Density (kg m^−3^)
- *ϕ*, Nanofluid volume fraction

Subscripts

- f, Fluid
- nf, Nanofluid
- s, Solid

## Additional file

Additional file 1:
**Cover letter to referee 3 and referee 4’s concerns.**

## References

[1] Choi SUS, Eastman JA. Enhancing thermal conductivity of fluids with nanoparticles. International mechanical engineering congress and exhibition, San Francisco, CA (United States), November 1995; Report number: ANL/MSD/CP-84938; CONF-951135-29 ON: DE96004174, TRN: 96:001707.

[2] Li Y, Zhou J, Tung S, Schneider E, Xi S (2009). A review on development of nanofluid preparation and characterization. Powder Technol.

[3] Jacopo B, David C (2009). Venerusetal: A benchmark study on the thermal conductivity of nanofluids. Journal of Applied Physics.

[4] Lazarus Godson B, Raja D, Mohan Lal S (2010). Wongwises: Enhancement of heat transfer using nanofluids - an overview. Renewable and Sustainable Energy Reviews.

[5] Ghadimi A, Saidur R, Metselaar HSC (2011). A review of nanofluid stability properties and characterization in stationary conditions. Int J Heat Mass Transf..

[6] Nsofor CE (2008). Recent patents on nanofluids (nanoparticles in liquids) heat transfer. Recent Patents Mechan Eng.

[7] Ostrach S (1988). Natural convection in enclosures. J Heat Transf.

[8] Wang X-Q, Mujumdar AS (2008). A review on nanofluids - part 2: experiments and applications. Braz J Chem Eng.

[9] Sarkar J (2011). A critical review on convective heat transfer correlations of nanofluids. Renew Sust Energ Rev..

[10] Kamyar A, Saidur R, Hasanuzzaman M (2012). Application of computational fluid dynamics (CFD) for nanofluids. Int J Heat Mass Transf..

[11] Haddad Z, Oztop HF, Abu-Nada E, Mataoui A (2012). A review on natural convective heat transfer of nanofluids. Renew Sust Energ Rev.

[12] Putra N, Roetzel W, Das SK (2003). Natural convection of nano-fluids. Heat Mass Transf.

[13] Wen D, Ding Y (2005). Formulation of nanofluids for natural convective heat transfer applications. Int J Heat Fluid Flow.

[14] Li CH, Peterson GP (2010). Experimental studies of natural convection heat transfer of Al_2_O_3_/DI water nanoparticle suspensions (nanofluids). Advances in Mechanical Engineering.

[15] Rui N, Sheng-Qi Z, Ke-Qing X (2011). An experimental investigation of turbulent thermal convection in water-based alumina nanofluid. Physics of Fluids.

[16] Nnanna AGA (2007). Experimental model of temperature-driven nanofluid. J Heat Transf.

[17] Ho CJ, Liu WK, Chang YS, Lin CC (2010). Natural convection heat transfer of alumina-water nanofluid in vertical square enclosures: an experimental study. Int J Therm Sci.

[18] Bresme F, Oettel M (2007). Nanoparticles at fluid interfaces. J Physics-Condense Matter..

[19] Lervik A, Bresme F, Kjelstrup S (2009). Heat transfer in soft nanoscale interfaces: the influence of interface curvature. Soft Matter..

[20] Eliodoro C, Pietro A (2011). Enhancing surface heat transfer by carbon nanofins: towards an alternative to nanofluids?. Nanoscale Research Letters.

[21] Eliodoro C, Matteo F, Pietro A, Paolo D (2014). Scaling behaviour for the water transport in nanoconfined geometries. Nature Communications.

[22] Khanafer K, Vafai K, Lightstone M (2003). Buoyancy-driven heat transfer enhancement in a two-dimensional enclosure utilizing nanofluids. Int J Heat Mass Transfer.

[23] Oztop HF, Abu-Nada E (2008). Numerical study of natural convection in partially heated rectangular enclosure filled with nanofluids. Int J Heat Fluid Flow.

[24] Aminossadati SM, Ghasemi B (2009). Natural convection cooling of a localised heat source at the bottom of a nanofluid-filled enclosure. European J Mechanics-B/Fluids.

[25] Ghasemi B, Aminossadati SM (2010). Brownian motion of nanoparticles in a triangular enclosure with natural convection. Int J Therm Sci.

[26] Fakhreddine Segni O, Rachid B (2011). Heterogeneous nanofluids: natural convection heat transfer enhancement. Nanoscale Research Letters.

[27] Ternik P, Rudolf R (2012). Heat transfer enhancement for natural convection flow of water-based nanofluids in a square enclosure. Int J Simulation Modelling.

[28] About OpenFOAM [http://www.openfoam.org/index.php]

[29] Instruction of Blender [http://www.blender.org/about/]

[30] Behzadmehr A, Saffar-Avval M, Galanis N (2007). Prediction of turbulent forced convection of a nanofluid in a tube with uniform heat flux using a two phase approach. Int J Heat Fluid Flow..

[31] Lotfi R, Saboohi Y, Rashidi AM (2010). Numerical study of forced convective heat transfer of nanofluids: comparison of different approaches. Int Comm Heat and Mass Transfer..

[32] Bianco V, Chiacchio F, Manca O, Nardini S (2009). Numerical investigation of nanofluids forced convection in circular tubes. Appl Therm Eng..

[33] Aminfar H, Motallebzadeh R, Farzadi A (2010). The study of the effects of thermophoretic and Brownian forces on nanofluid thermal conductivity using Lagrangian and Eulerian approach. Nanos Microscale Thermophysical Eng..

[34] Aminfar H, Mohammad RH (2012). Brownian motion and thermophoresis effects on natural convection of alumina-water nanofluid. J Mech Eng Sci.

[35] Akbari M, Galanis N, Behzadmehr A (2012). Comparative assessment of single and two-phase models for numerical studies of nanofluid turbulent forced convection. Int J Heat Fluid Flow..

[36] Mostafa Keshavarz M, Elahe E (2012). Comparison between single-phase and two-phases CFD modeling of laminar forced convection flow of nanofluids in a circular tube under constant heat flux. International Communications in Heat and Mass Transfer.

[37] Wen D, Lin G, Vafaei S, Zhang K (2009). Review of nanofluids for heat transfer applications. Particuol.

[38] Wenhua Y, France DM, Jules L, Routbort, Stephen U S C (2008). Review and comparison of nanofluid thermal conductivity and heat transfer enhancements. Heat Transfer Engineering.

[39] OpenFOAM programmer’s guide [http://www.openfoam.org/docs/user/]

[40] Boussinesq J: Theorie Analytique de la Chaleur. Gauthier-Villars; 1903.

[41] John H Lienhard IV, John H Lienhard V: A heat transfer textbook (third edition). Phlogiston Press; 2008.

[42] Sarit Kumar D, Nandy P, Peter T, Wilfried R (2003). Temperature dependence of thermal conductivity enhancement for nanofluids. Journal of Heat Transfer.

[43] Botte GG, Ritter JA, White RE (2000). Comparison of finite difference and control volume methods for solving differential equations. Comput Chem Eng..

[44] Ferziger JH, Peric M (2002). Computational methods for fluid dynamics (vol. 3).

[45] enGrid - open-source mesh generation [http://engits.eu/en/engrid]

[46] Corzo SF, Damián SM, Ramajo D, Nigro NM (2011). Numerical simulation of natural convection phenomena. Mecánica Computacional..

[47] Sanjeeva W, Chris H, Dan X, Xiaojun L, Yulong D (2012). Aggregation and settling in aqueous polydisperse alumina nanoparticle suspensions. J Nanoparticle Res.

[48] Welcome to ParaView [http://www.paraview.org/]

[49] Biswas G, Breuer M, Durst F (2004). Backward-facing step flows for various expansion ratios at low and moderate Reynolds numbers. Journal of Fluids Engineering.

[50] Armaly BF, Durst F, Pereira JCF, Schonung B (1983). Experimental and theoretical investigation of backward-facing step flow. J Fluid Mech..

[51] Barakos G, Mitsoulis E (1994). Natural convection flow in a square cavity revisited: laminar and turbulent models with wall functions. Int J Numer Methods Fluids.

