# Supplementary material for: Numerical study of natural convection in a horizontal cylinder filled with water-based alumina nanofluid
Source: Nanoscale Res Lett. 2015 Mar 19;10:142. doi: 10.1186/s11671-015-0847-x (PMC4385295; doi:10.1186/s11671-015-0847-x)
Supplement: Additional file 1: — Cover letter to referee 3 and referee 4’s concerns. [file 11671_2015_847_MOESM1_ESM.pdf]

Cover letter to Referee3 and Referee 4’s concerns

Dear Editorial board of Nanoscale Research Letter:

First of all, really appreciate your patience and all the things you did for our manuscript.

According to your kindly advice, we have made following point-to-point revisions and replies regarding to Refree3 and Refree4’s concerns. Furthermore, for your convenience, all the references to support our points are attached at the end this cover letter.

Referee3’s comments and our thoughts

Comment1: Actually, I still believe that a single-phase model cannot be used to give a reasonable explanation of what happens in enclosed natural convection of nanofluids, which makes the paper not interesting.

Our thoughts: Today, single-phase approach is one of the most popular methods for nanofluid flow and heat transfer CFD simulation. A good summary table for recent CFD studies can be found in [1], from which it can be found that more than 70% CFD simulations are carried out with single-phase approach. Although some CFD investigations have been published based on Mixture, VOF, Eulerian-Eulerian and Eulerian-Lagrangian approaches recently[2-5], however by Bianco’s study[6], single- and multi-phase models present very similar results in their work. In Akbari’s investigation[7], it shows that single-phase approach is even better than multi-phase approache to give good agreement with experimental study.

Comment2: On the other hand, the fact that "the mechanisms of nanofluid thermal conductivity enhancement are still not very clear" and that in the present work "the essential nanofluid properties (thermal conductivity and viscosity) are

collected from experimental investigations instead of prediction models" has nothing to do with the use of a single or two-phase models.

Our thoughts: "The mechanisms of nanofluid thermal conductivity enhancement are still not very clear" is a commonly accepted conclusion in nanofluid research[8, 9]. So far, no paper can be found to disclose the reasons for sure why nanofluid has such a good heat transfer performance enhancement[9, 10]. Currently, nearly all prediction models for nanofluid thermal conductivity and viscosity are proposed based on some assumptions[11, 12], e.g. nanoparticles are spherical[13] or cylindrical[14]. That is the reason why we use the data (regression data) collected from experimental investigations instead of those prediction models which may cause inaccurate simulation. According to the experimental data, our numerical solver updates the non-uniform nanofluid properties in the whole computational region after each iteration to make the simulation more practical. This is the way how do we use the experimental data.

Referee4's comments and our revisions

Compulsory Revisions

1. Show component names and legends in Figure 1. For instance, Tc = cooling temperature; D = cylinder diameter, etc.

   Revision: The meanings of these components are introduced in session '2.1 Problem configuration'. They can also be found in 'Nomenclature' at the end of paper.

2. On page 6, Cp should be "specific heat at constant pressure".

   Reason for no revision: Nanofluid is considered as incompressible fluid as water, specific heat property is default as a stable value without variation induced by pressure change.

3. Units on page 7, "Kg" should be "kg" and "k" should be "K" (absolute temperature). On the same page, give an explanation why the values of "3970 kg/m3 and 765 J/kg.K" are used in the study other than other values. Viscosity v differs with dynamic viscosity, which needs to be defined. In addition, (T-273) is to

change absolute temperature to Celsius scale, which should give units or explanation.

Revision: Corrected "Kg" to "kg" and "k" to "K" on page 7. A note is given for ‘Celsius scale’ on page 7. 3970 kg/m^3 and 765 $J/kg \cdot K$ are the most commonly accepted parameters for Al_2O_3 nanoparticle properties. Viscosities are defined with ‘dynamic’ and ‘kinetic’ on page 7 and 8. The explanation of (T-273) is given on page 7 as ‘Based on regression analysis from Das’s study[15, 16]’.

4. On page 8, "heat transfer coefficient" should be convection heat transfer coefficient". Units of the parameters h, Lc, etc. should be given.

   Revision: "heat transfer coefficient" has been corrected to "convection heat transfer coefficient" on page8. Units of the parameters h, Lc, etc are defined in ‘Nomenclature’ at the end of this paper.

5. On page 11, what is the definition of X, section area or length?

   Revision: The definition of ‘X’ is added on page 11 as ‘cylinder cross section position’.

6. What is the conclusion of this study: Heat Transfer Deterioration or Enhancement? In Abstract, the conclusion is not given either. Readers will interested in the study results, not numerical study itself.

   Revision: ‘At same Rayleigh number, Nusselt number is found to decrease with nanofluid volume fraction.’ can be found in abstract, as well as the similar conclusion in the main body of this paper on page 12. In other word, heat transfer deterioration is found in our present work.

Minor Essential Revisions:

1. Symbols and words need to be used consistently. For instance, volume fraction and #, the paper uses #, volume fraction, and volume fraction # for the same purpose.

   Revision: Correlative corrections have been made on page 2.

2. The second paragraph and third paragraph in Introduction are better to be tabulated. They are the description of previous studies. it is not the main discussion of this paper. Otherwise, these two paragraphs seem a little bit boring due to repeatedly the same description format. Table is concise and early to be read. Columns can be "Author", "Experiment", "Observation", etc.

   Reason for no revision: We still think it is necessary to provide readers two narrative paragraphs before introducing our idea. Perhaps it is a bit boring, but it could be better to make the whole paper more consistent.

3. English and typo. Page 3, "numerical study" should be "numerical studies". Page 5, "insulate cylinder" should be "insulated cylinder" and "D=0.04m, respectively". Eliminate "water", the sentence should be "natural heat convection of 1% Al2O3 and 4%..." Page 21, "Prof" should be "Prof."

   Revision: Corrected "numerical study" to "numerical studies" on page 3. Corrected "insulate cylinder" to "insulated cylinder" on page 5. Corrected "D=0.04m respectively" to "D=0.04m, respectively" on page 5. Corrected "Prof" to "Prof." on page 21.

4. On page 6, units for the parameters T, k, ..., should be shown. U (velocity) and P (pressure) should be in lowercase. Also, use "gradient operations, respectively" and other areas too.

   Revision: The units for the parameters T, k, ... are given in ‘Nomenclature’ at the end of this paper. Corrected "gradient operations respectively" to "gradient operations, respectively" on page 6.

Reference to support our points

[1] A. Kamyar, R. Saidur, M. Hasanuzzaman, Application of computational fluid dynamics (CFD) for nanofluids, International Journal of Heat and Mass Transfer, 55 (2012) 4104-4115.
[2] M. Kalteh, A. Abbassi, M. Saffar-Avval, A. Frijns, A. Darhuber, J. Harting, Experimental and numerical investigation of nanofluid forced convection inside a wide microchannel heat sink, Applied Thermal Engineering, 36 (2012) 260-268.
[3] A. Behzadmehr, M. Saffar-Avval, N. Galanis, Prediction of turbulent forced convection of a nanofluid in a tube with uniform heat flux using a two phase approach, International Journal of Heat and Fluid Flow, 28 (2007) 211-219.

[4] R. Lotfi, Y. Saboohi, A.M. Rashidi, Numerical study of forced convective heat transfer of nanofluids: comparison of different approaches, International Communications in Heat and Mass Transfer, 37 (2010) 74-78.
[5] M.K. Moraveji, R.M. Ardehali, CFD modeling (comparing single and two-phase approaches) on thermal performance of Al 2 o 3/water nanofluid in mini-channel heat sink, International Communications in Heat and Mass Transfer, 44 (2013) 157-164.
[6] V. Bianco, F. Chiacchio, O. Manca, S. Nardini, Numerical investigation of nanofluids forced convection in circular tubes, Applied Thermal Engineering, 29 (2009) 3632-3642.
[7] M. Akbari, N. Galanis, A. Behzadmehr, Comparative assessment of single and two-phase models for numerical studies of nanofluid turbulent forced convection, International Journal of Heat and Fluid Flow, 37 (2012) 136-146.
[8] P. Keblinski, S.R. Phillpot, S.U.S. Choi, J.A. Eastman, Mechanisms of heat flow in suspensions of nano-sized particles (nanofluids), International Journal of Heat and Mass Transfer, 45 (2002) 855-863.
[9] J. Buongiorno, D.C. Venerus, N. Prabhat, T. McKrell, J. Townsend, R. Christianson, Y.V. Tolmachev, P. Keblinski, L.-w. Hu, J.L. Alvarado, A benchmark study on the thermal conductivity of nanofluids, Journal of Applied Physics, 106 (2009) 094312.
[10] Y. Li, S. Tung, E. Schneider, S. Xi, A review on development of nanofluid preparation and characterization, Powder Technology, 196 (2009) 89-101.
[11] Y. Xuan, Q. Li, W. Hu, Aggregation structure and thermal conductivity of nanofluids, AIChE Journal, 49 (2003) 1038-1043.
[12] W. Evans, R. Prasher, J. Fish, P. Meakin, P. Phelan, P. Keblinski, Effect of aggregation and interfacial thermal resistance on thermal conductivity of nanocomposites and colloidal nanofluids, International Journal of Heat and Mass Transfer, 51 (2008) 1431-1438.
[13] M. Izadi, S. Hossainpour, V.D. Jalali, Effects of nanolayer structure and Brownian motion of particles in thermal conductivity enhancement of nanofluids, Int J Mech Ind Aerosp Eng, 3 (2009) 201.
[14] S. Ebrahimi, J. Sabbaghzadeh, M. Lajvardi, I. Hadi, Nanolayer effects in thermal conductivity of nanofluids containing cylindrical nanoparticles, in: Nanotechnology, 2007. IEEE-NANO 2007. 7th IEEE Conference on, IEEE, 2007, pp. 1195-1198.
[15] S.K. Das, N. Putra, P. Thiesen, W. Roetzel, Temperature dependence of thermal conductivity enhancement for nanofluids, Journal of Heat Transfer, 125 (2003) 567-574.
[16] N. Putra, W. Roetzel, S.K. Das, Natural convection of nano-fluids, Heat and Mass Transfer, 39 (2003) 775-784.
